# Risk Factors Associated with Severe Disease and Intensive Care Unit Admission of Pregnant Patients with COVID-19 Infection—A Retrospective Study

**DOI:** 10.3390/jcm11206055

**Published:** 2022-10-13

**Authors:** Ana-Maria Adam, Ingrid-Andrada Vasilache, Demetra Socolov, Mariana Stuparu Cretu, Costinela Valerica Georgescu, Petronela Vicoveanu, Elena Mihalceanu, Anamaria Harabor, Razvan Socolov

**Affiliations:** 1 Clinical and Surgical Department, Faculty of Medicine and Pharmacy, ‘Dunarea de Jos’ University, 800216 Galati, Romania; 2 Department of Obstetrics and Gynecology, ‘Grigore T. Popa’ University of Medicine and Pharmacy, 700115 Iasi, Romania; 3 Medical Department, Faculty of Medicine and Pharmacy, ‘Dunarea de Jos’ University, 800216 Galati, Romania; 4 Department of Pharmaceutical Sciences, Faculty of Medicine and Pharmacy, ‘Dunarea de Jos’ University, 800216 Galati, Romania

**Keywords:** COVID-19, SARS-CoV-2, pregnancy, intensive care unit, risk factors

## Abstract

(1) Background: Pregnant patients with severe forms of coronavirus disease 2019 (COVID-19) can experience adverse pregnancy outcomes. The aim of this study was to retrospectively assess the risk factors associated with admission to the intensive care unit (ICU) of pregnant patients with COVID-19, as well as the pregnancy outcomes of these patients; (2) Methods: Medical records of 31 pregnant patients with COVID-19 admitted to three clinical hospitals from Romania, between October 2020 and November 2021 were examined. The patients were segregated into two groups depending on their clinical evolution: non-ICU admission (*n* = 19) or ICU admission (*n* = 12). Clinical and paraclinical findings were evaluated using univariate analysis, and the association of significant risk factors with maternal ICU admission was assessed using a multivariate analysis. Pregnancy outcomes of these patients were also recorded; (3) Results: Pulmonary disease, cough, dyspnea, leukocytosis, thrombocytosis, high serum values of transaminases, serum ferritin, and increased duration of hospital admission were identified as significant risk factors associated with maternal admission to the ICU. No significant differences regarding pregnancy outcomes were noted between the evaluated patients; (4) Conclusions: Specific risk factor identification in pregnant patients with severe forms of COVID-19 could improve the patient’s management.

## 1. Introduction

The SARS-CoV-2 infection is associated with significant maternal morbidity and mortality [1]. Recent studies suggest that pregnant women with SARS-CoV-2 infection, also known as coronavirus disease 2019 (COVID-19), have an increased risk of intensive care unit (ICU) admission, mechanical ventilation, and death when compared to pregnant women without this type of disease [2,3]. Therefore, it is important to promptly identify the patients who are at increased risk for developing severe complications in order to provide the best management of the case, and to prevent long-term consequences.

The estimated mortality rates for pregnant patients with COVID-19 varies between 1.35% and 12.3% [4,5,6]. The mortality rates are influenced by vaccinal status and individual clinical risk factors [7,8,9] Pregnancy and puerperal periods represent a particular clinical context due to hormonal and immunological specific changes, and specific effects of new vaccines against SARS-CoV-2 infection were not studied during their pre-marketing trials [10]. However, the post-marketing evidence of efficacy and safety have shown a good risk-benefit profile of these vaccines [10], and therefore most scientific societies recommend COVID-19 vaccination during pregnancy and breastfeeding [11,12].

Regarding the individual risk factors that influence the mortality rates of pregnant patients with COVID-19, a recent meta-analysis evaluated the influence of maternal diabetes, obesity, and asthma, and concluded that maternal obesity increased the risk of maternal death by 2.48 (95% CI 1.41–4.36), while the presence of one risk factor increased the risk of death two-fold during COVID-19 infection (risk ratio (RR): 2.26, 95% confidence interval (CI): 1.77–2.89) [13]. Moreover, another meta-analysis calculated—using a random effects model—the effect size of comorbidities on maternal mortality in COVID-19 patients, and demonstrated an effect size for obesity of 0.47 (95% CI: 0.04–0.90, *p* = 0.03), 0.29 for diabetes (95% CI: −0.08–0.65, *p* = 0.12), 0.41 for asthma (95% CI: −0.06–0.88, *p* = 0.09), and 0.61 for advanced maternal age (95% CI: 0.13–1.08, *p* = 0.01) [14].

A recent systematic review and meta-analysis proved that pregnant women are more prone to be admitted to an ICU (odds ratio (OR): 1.62, 95% CI: 1.33–1.96) and require more invasive ventilation (OR: 1.88; 95% CI: 1.36–2.60) as compared to non-pregnant women [3]. However, risk factors for ICU admission of pregnant patients diagnosed with COVID-19 are poorly explored. A retrospective multicenter study evaluated the management and outcomes of 187 pregnant women with COVID-19 admitted to several ICUs from Europe, and identified as risk factors for intubation obesity (cause-specific hazard ratio (CSH): 2.00, 95% CI: 1.05–3.80), *p*  =  0.03), term of pregnancy (CSH: 1.07, 95% CI: 1.02–1.10), per  +  1 week gestation, *p* =  0.01), extent of computed tomography (CT) scan abnormalities  >  50% (CSH: 2.69, 95% CI: 1.30–5.60), *p* <  0.01) and non-invasive ventilation (NIV) use (CSH 2.06, 95% CI (1.09–3.90), *p* =  0.03) [15]. A recent case-control study showed that gestational diabetes also presented with a higher rate of ICU admission in SARS-CoV-2 pregnant patients [16].

Moreover, a multicentric retrospective cohort study developed a predictive model for the admission of 44 pregnant patients to the ICU which included body mass index (OR: 1.39; 95% CI: 1.07–1.95; *p* = 0.015), lower respiratory symptoms (OR: 5.11; 95% CI: 1.81–21.4; *p* = 0.007), neutrophil to lymphocyte ratio (OR: 1.62; 95% CI: 1.36–1.89; *p* < 0.001); and serum C-reactive protein (OR: 1.30; 95% CI: 1.15–1.44; *p* < 0.001), and had an adjusted area under the receiver operating characteristic curve of 0.85 [17].

Limited data exists in the current literature regarding the clinical determinants of COVID-19 severity in hospitalized pregnant women. The aim of this study was to retrospectively assess the risk factors associated with severe disease and ICU admission of pregnant patients with COVID-19, as well as their pregnancy outcomes.

## 2. Materials and Methods

We conducted an observational retrospective study of pregnant patients with COVID-19 admitted to three clinical hospitals: the Obstetrics and Gynecology Hospital Buna Vestire, Galati, Elena–Doamna Maternity Hospital, Iasi, and Saint John Emergency Hospital, Suceava, Romania, between October 2020 and November 2021. All patients were initially evaluated in the obstetrics and gynecology departments of these hospitals. The SARS-CoV-2 infection was confirmed after the evaluation of nasopharyngeal swabs using the polymerase chain reaction (PCR) assay. The main circulating variants of SARS-CoV-2 were Alpha, for the October 2020–September 2021 time frame, and Delta, for the October–November 2021 time frame [18,19]. Admission to the ICU was at the discretion of the consulted critical care attending physician at each site.

Ethical approval for this study was obtained from the Institutional Ethics Committees of University of Medicine and Pharmacy Grigore T. Popa (No. 27/04.01.2021), which applies to the research activity from Elena–Doamna Maternity Hospital, Saint John Emergency Hospital, and Buna Vestire Obstetrics and Gynecology Hospital (No. 6793/08.09.2020). Informed consent was obtained from all participants included in the study. All methods were carried out in accordance with relevant guidelines and regulations.

Medical records of patients were systematically reviewed and data were obtained. Exclusion criteria comprised patients who had multiple pregnancies, ectopic pregnancies, first and second trimester abortions, fetal intrauterine demise, fetuses with chromosomal or structural abnormalities, intrauterine infection, incomplete medical records, or who were unable to offer informed consent due to various reasons (age less than 18 years old, intellectual deficits, psychiatric disorders, etc.).

31 patients were segregated into two groups depending on their clinical evolution: non-ICU admission (*n* = 19) or ICU admission (*n* = 12). The following variables were recorded: demographic data, the patient’s medical history, clinical manifestations, laboratory parameters at admission, imaging findings, pregnancy outcomes, length of ICU stay, length of hospital stay, the need for invasive mechanical ventilation, and the treatment received.

Statistical analysis was performed using Stata SE (version 15, StataCorp, College Station, TX, USA). Bivariate associations between the status at discharge status and each risk factor were evaluated with chi-square and Fisher’s exact tests for categorical variables, and *t*-test for continuous variables. A *p* value of less than 0.05 was considered to be statistically significant. Variables with a significant *p* value from the univariate analysis were entered into a multivariate analysis.

## 3. Results

This retrospective study evaluated 31 pregnant patients diagnosed with COVID-19. The clinical characteristics of the evaluated groups and the results from the univariate analysis are presented in Table 1. We found a statistically significant difference between the two groups regarding their personal history of pulmonary disease (*p* = 0.012) and obesity (*p* = 0.038). Considering the personal history of pulmonary disease, one patient (5.3%), who was not admitted to the ICU, had childhood tuberculosis, while 4 patients (33.3%) who were admitted to the ICU, had chronic obstructive pulmonary disease due to smoking, and one patient (8.3%) had a mild form of asthma.

Among the clinical manifestations of these patients, cough (10 patients; 83.3%) and dyspnea (10 patients; 83.3%) were significantly more frequently encountered in the group of patients who were admitted to the ICU (*p* < 0.05). At the same time, the imaging findings (computed tomography and chest radiography) revealed significantly more ground glass opacities (7 patients; 58.3%; *p* = 0.014) and patchy consolidations (5 patients; 41.7%; *p* = 0.043) in the group of patients who had a severe form of COVID-19 and were admitted to the ICU (Table 2).

The univariate analysis of the main laboratory parameters revealed a significantly higher frequency of inflammatory syndrome (leukocytosis, elevated C-reactive protein and ferritin) and hepatic cytolysis (elevated transaminases) in the group of patients who were admitted to the ICU (*p* < 0.05) (Table 3).

The univariate analysis of the respiratory interventions conducted during hospital stay indicated that both means of ventilation (mechanical and non-invasive) were significantly more used in the second group (*p* < 0.001). Moreover, the mean length of hospital stay was significantly higher for the group of patients who were admitted to the ICU (14.58 versus 8.57 days, *p* < 0.001) (Table 4).

The multivariate analysis showed a significant association between personal history of pulmonary disease (OR: 8.76, 95%CI: 0.70–109.24, *p* = 0.039), cough (OR: 8.43, 95%CI: 1.25–56.64, *p* = 0.028), dyspnea (OR: 0.06, 95%CI: 0.008–0.50, *p* = 0.009), leukocytosis (OR: 0.99, 95%CI: 0.99–1.01, *p* = 0.012), thrombocytosis (OR: 1.04, 95%CI: 1.00–1.05, *p* = 0.007), high serum values of TGO (OR: 0.80, 95%CI: 0.69–0.94, *p* = 0.008), TGP (OR: 0.89, 95%CI: 0.81–0.97, *p* = 0.01), ferritin (OR: 0.96, 95%CI: 0.991–0.998, *p* = 0.002), increased duration of hospital stay (OR: 1.38, 95%CI: 1.06–1.81, *p* = 0.016), and intensive care admission (Table 5).

None of the patients were vaccinated against COVID-19 and no deaths were recorded. All patients admitted to the ICU received treatment with an antiviral (Remdesivir, Lopinavir/Ritonavir, or Umfenovir), thromboprophylaxis with low molecular weight heparin (LMWH), and antibiotic therapy. The antiviral of choice for our pregnant patients admitted to the ICU was Remdesivir (8 patients, 66.6%), while the combination between Lopinavir/Ritonavir was administered in 3 cases (25%), and Umfenovir in a single case (8.33%).

The interaction analysis between categorical variables included in the multivariate analysis is represented in Table 6. None of the interactions between the evaluated parameters had a statistical significance.

Our results failed to indicate a statistically significant difference between the two groups regarding the pregnancy outcomes: gestational age at birth (*p* = 0.13), birth weight (*p* = 0.56), Apgar score 5 min (*p* = 0.73), sex (*p* = 0.47), mode of delivery (*p* = 0.61), premature rupture of membranes (*p* = 0.63), and preterm labour before 37 weeks of gestation (*p* = 0.67), respectively (Table 7).

## 4. Discussion

In this retrospective study, we assessed the risk factors associated with maternal admission to the intensive care units of three hospitals from Romania.

Our univariate analysis indicated that a personal history of pulmonary disease and obesity was significantly more frequently encountered in the group of patients who were admitted to the ICU. A preliminary analysis of maternal mortality during the COVID-19 pandemic in Mexico indicated that a high body mass index (BMI) increased the odds for death (OR: 1.70), while pulmonary disease was associated with an immune impairment [20].

Furthermore, cough (10 patients; 83.3%) and dyspnea (10 patients; 83.3%) were significantly more frequently encountered in the group of patients who had a severe form of COVID-19, and required intensive monitorization. Our results are in line with those cited in an observational study that described the clinical characteristics of 447 maternal deaths due to severe forms of COVID-19, and revealed that the most frequent symptoms at first consultation and admission were dyspnea (73%), fever (69%), and cough (59%) [21].

Imaging findings indicated a significantly higher prevalence of ground glass opacities (7 patients; 58.3%; *p* = 0.014) and patchy consolidations (5 patients; 41,7%; *p* = 0.043) in the group of patients who necessitated intensive care, while the laboratory findings indicated a significantly higher frequency of inflammatory syndrome (leukocytosis, elevated C-reactive protein and ferritin) and hepatic cytolysis (elevated transaminases) in this group of patients.

A systematic review by Oshay et al. evaluated the literature data from 67 articles regarding imaging findings in chest CT scans and associated clinical features in pregnant patients diagnosed with COVID-19. The authors discovered that ground-glass opacities (77.2%, 250/324) and consolidation (40.9%, 94/230) were frequently encountered among pulmonary findings in chest CT scans, and that pregnant patients presented more frequently with consolidation (40.9% vs. 21.0–31.8%) in comparison to the general population [22].

In a recent retrospective study, the authors concluded that high ferritin, and C-reactive protein serum values were associated with poor prognosis and mortality in unvaccinated pregnant women with SARS-CoV-2 infection [23]. Moreover, a cross-sectional study that evaluated the clinical course of maternal mortality cases due to severe forms of COVID-19 indicated a higher frequency of leukocytosis with raised neutrophils: lymphocytes ratio, thrombocytopenia and elevated levels of acute phase reactants and inflammatory markers such as CRP, serum ferritin, lactate dehydrogenase (LDH), D-dimer, and serum fibrinogen in their cohort of unvaccinated patients [24].

Our univariate analysis of the respiratory interventions conducted during hospital stay indicated that both means of ventilation (mechanical and non-invasive) were significantly more used in the second group, while the mean length of hospital stay was significantly higher for those patients. A recent retrospective study assessed maternal and neonatal outcomes of 19 critically ill pregnant and puerperal patients in the clinical course of COVID-19, and the authors reported that 8 patients died, all of whom received mechanical ventilation, resulting in an ICU mortality rate of 42.1% [25]. Additionally, the authors discovered that the mean number of hospitalized days in ICU was significantly lower in patients who were discharged (*p* = 0.037).

The multivariate analysis showed a significant association between pulmonary disease, respiratory symptoms, and certain specific serum markers with intensive care unit admission, and our results are supported by data from systematic reviews and meta-analyses [3,13,26,27,28,29,30]. Moreover, a recent secondary analysis of a national prospective cohort study that included 4436 pregnant patients confirmed the association of maternal obesity (OR: 2.52 95% CI: 1.97–3.23) and gestational diabetes (OR: 1.43, 95% CI: 1.09–1.87) with severe forms of COVID-19 [31].

Although we could not demonstrate a statistically significant difference between the two groups regarding the pregnancy outcomes, several studies outlined higher rates of preterm births, stillbirth, cesarean deliveries, and admissions to the neonatal intensive care units for pregnancies affected by severe forms of COVID-19 [31,32,33]. We hypothesized that, due to our small cohort of patients, a significant association between severe forms of COVID-19 and adverse pregnancy outcomes could not be emphasized.

One of the limitations of our study is represented by the small sample size, which can constitute a selection bias. The retrospective design of this study is another limitation, and we consider that a prospective design would offer stronger evidence over the association of specific risk factors with ICU admission. Finally, the heterogeneity of the clinical and paraclinical findings constitutes a limitation in the relationship with the above-mentioned caveats. A greater cohort of patients recruited from multiple centers would allow for a more comprehensive picture of the issue.

Specific risk factor identification in pregnant patients with severe forms of COVID-19 could allow for individualized patient management, which clinicians can then put to use in order to make the best therapeutic decisions.

## 5. Conclusions

The COVID-19 pandemic constituted a real challenge for the clinicians in terms of patient’s management. The pregnancy itself can be accompanied by unforeseeable risks and complications, making the risk stratification process even more difficult for pregnant patients with COVID-19. Therefore, it is crucial to identify all clinical and paraclinical parameters that would ease this process. The results of this study could be taken into consideration by clinicians for the management of pregnant patients with severe forms of COVID-19. 

## Figures and Tables

**Table 1:** A univariate analysis of the clinical characteristics for the patients included in our study.

Patient’s Data		Non-ICU Admission (*n* = 19 Patients)	ICU Admission (*n* = 12 Patients)	*p* Value
Demographics	Maternal age, years (mean and standard deviation)	26.21 ± 5.19	27.5 ± 3.42	0.22
	Medium (*n*/%)	Rural = 7 (36.8%)	Rural = 5 (63.2%)	0.54
		Urban = 12 (63.2%)	Urban = 7 (58.3%)	
Clinical parameters	Preeclampsia (*n*/%)	No = 19 (100%)	No = 10 (83.3%	0.14
		Yes = 0 (0%)	Yes = 2 (16.7%)	
	Gestational diabetes (*n*/%)	No = 18 (94.7%)	No = 10 (83.3%	0.54
		Yes = 1 (5.3%)	Yes = 2 (16.7%)	
	Obesity (*n*/%)	No = 18 (94.7%)	No = 8 (66.7%)	0.038
		Yes = 1 (5.3%)	Yes = 4 (33.3%)	
	Pulmonary disease (*n*/%)	No = 18 (94.7%)	No = 7 (58.3%)	0.012
		Yes = 1 (5.3%)	Yes = 5 (41.7%)	
	Smoking (*n*/%)	No = 17 (89.5%)	No = 8 (66.7%)	0.117
		Yes = 2 (10.5%)	Yes = 4 (33.3%)	
	Gestation (*n*/%)	Primigravida = 12 (63.2%)	Primigravida = 6 (50.0%)	0.46
		Multigravida = 7 (36.8%)	Multigravida = 6 (50%)	
	Parity (*n*/%)	Primipara = 17 (89.5%)	Primipara = 8 (66.7%)	0.11
		Multipara = 2 (10.5%)	Multipara = 4 (33.3%)	

ICU—intensive care unit.

**Table 2:** A univariate analysis of the clinical manifestations and imaging findings for patients included in our study.

Parameters Evaluated		Non-ICU Admission (*n* = 19 Patients)	ICU Admission (*n* = 12 Patients)	*p* Value
Clinical manifestations	Fever (*n*/%)	No = 9 (47.4%)	No = 3 (25.0%)	0.21
		Yes = 10 (52.6%)	Yes = 9 (75.0%)	
	Cough (*n*/%)	No = 13 (68.4%)	No = 2 (16.7%)	0.005
		Yes = 6 (31.6%)	Yes = 10 (83.3%)	
	Myalgia (*n*/%)	No = 15 (78.9%)	No = 8 (66.7%)	0.44
		Yes = 4 (21.1%)	Yes = 4 (33.3%)	
	Headache (*n*/%)	No = 16 (84.2%)	No = 9 (75.0%)	0.52
		Yes = 3 (15.8%)	Yes = 3 (25.0%)	
	Dyspnea (*n*/%)	No = 15 (78.9%)	No = 2 (16.7%)	0.001
		Yes = 4 (21.1%)	Yes = 10 (83.3%)	
	Anosmia/ageusia (*n*/%)	No = 16 (84.2%)	No = 9 (75.0%)	0.52
		Yes = 3 (15.8%)	Yes = 3 (25.0%)	
Imaging findings	Ground glass opacities	No = 16 (84.2%)	No = 5 (41.7%)	0.014
		Yes = 3 (15.8%)	Yes = 7 (58.3%)	
	Patchy consolidation	No = 17 (89.5%)	No = 7 (58.3%)	0.043
		Yes = 2 (10.5%)	Yes = 5 (41.7%)	
	Reticular pattern	No = 17 (89.5%)	No = 8 (66.7%)	0.11
		Yes = 2 (10.5%)	Yes = 4 (33.3%)	

ICU—intensive care unit.

**Table 3:** A univariate analysis of the laboratory parameters for patients included in our study.

Laboratory Parameters	Non-ICU Admission (*n* = 19 Patients)	ICU Admission (*n* = 12 Patients)	*p* Value
Leucocytes/mm^3^ (mean, standard deviation, and 95%CI)	16052.63 ± 2655.67 (14772.64–17332.63)	21333.33 ± 5761.68 (17672.54–24994.13)	0.001
Thrombocytes/mm^3^ (mean, standard deviation, and 95%CI)	156631.6 ± 58910.68 (128237.5–185025.6)	87333.33 ± 25867.96 (70897.61–103769.1)	<0.001
C−reactive protein, mg/dL (mean, standard deviation, and 95%CI)	1.47 ± 0.29 (1.32–1.6)	5.56 ± 3.95 (3.05–8.07)	<0.001
Glutamic-oxaloacetic transaminase (TGO), U/L (mean, standard deviation, and 95%CI)	22.31 ± 6.82 (19.02–25.60)	36.33 ± 10.95 (29.37–43.29)	<0.001
Glutamic pyruvic transaminase (TGP), U/L (mean, standard deviation, and 95%CI)	18.68 ± 1.53 (15.46–21.90)	35.91 ± 17.44 (24.83–46.99)	<0.001
Ferritin, ng/mL (mean, standard deviation, and 95%CI)	620.05 ± 248.89 (500.08–740.01)	1344.58 ± 444.92 (1061.88–1627.27)	<0.001

ICU—intensive care unit, CI—confidence interval, TGO—Glutamic-oxaloacetic transaminase, TGP—Glutamic pyruvic transaminase.

**Table 4:** A univariate analysis of the parameters evaluated during hospital stay for the patients included in our study.

Parameters Evaluated	Non-ICU Admission (*n* = 19 Patients)	ICU Admission (*n* = 12 Patients)	*p* Value
Mechanical ventilation (*n*/%)	No = 19 (100%)	No = 7 (58.3%)	<0.001
	Yes = 0 (0%)	Yes = 5 (41.6%)	
Non-invasive ventilation (*n*/%)	No = 17 (89.4%)	No = 5 (41.6%)	<0.001
	Yes = 2 (10.5%)	Yes = 7 (58.3%)	
Length of hospital stay, days (mean, standard deviation, and 95%CI)	8.57 ± 2.73 (7.26–9.89)	14.58 ± 6.58 (10.39–18.76)	<0.001

ICU—intensive care unit, CI—confidence interval.

**Table 5:** A multivariate analysis of the parameters associated with maternal admission to the intensive care unit.

Parameters Evaluated	Odds Ratio	95%CI	*p* Value
Obesity	0.11	0.01–1.15	0.06
Pulmonary disease	8.76	0.70–109.24	0.039
Cough	8.43	1.25–56.64	0.028
Dyspnea	0.06	0.008–0.50	0.009
Ground glass opacities	0.15	0.01–2.10	0.16
Patchy consolidation	0.8	0.04–14.64	0.88
Leucocytes	0.99	0.99–1.01	0.012
Thrombocytes	1.04	1.00–1.05	0.007
Length of hospital stay	1.38	1.06–1.81	0.016
High TGO serum levels	0.80	0.69–0.94	0.008
Glutamic pyruvic transaminase (TGP)	0.89	0.81–0.97	0.01
Ferritin	0.96	0.991–0.998	0.002

ICU—intensive care unit, CI—confidence interval, TGO—Glutamic-oxaloacetic transaminase, TGP—Glutamic pyruvic transaminase.

**Table 6:** An interaction analysis between the symptoms and imaging findings included in the multivariate analysis.

Parameters Evaluated	*p* Value	95%CI
Pulmonary disease * cough	0.22	−0.58–0.14
Pulmonary disease * dyspnea	0.13	−0.12–0.84
Pulmonary disease * ground glass opacities	0.089	−0.81–1.08
Pulmonary disease * patchy consolidation	0.065	−0.55–1.72
Cough * dyspnea	0.083	−0.07–1.20
Cough * ground glass opacities	0.60	−0.36–0.61
Cough * patchy consolidations	0.83	−0.58–047
Dyspnea * ground glass opacities	0.34	−0.78–0.28
Dyspnea * patchy consolidations	0.28	−0.83–0.25
Ground glass opacities * patchy consolidations	0.43	−0.90–0.40

* symbol for interaction between variables.

**Table 7:** The pregnancy outcomes for the two groups of patients included in the study.

Pregnancy Outcomes	Non-ICU Admission (*n* = 19 Patients)	ICU Admission (*n* = 12 Patients)	*p* Value
Gestational age, weeks (mean, standard deviation, and 95%CI)	38.47 ± 1.71 (37.64–39.29)	37.5 ± 1.78 (36.36–38.63)	0.13
Birth weight, g (mean, standard deviation, and 95%CI)	3170 ± 549.49 (2905.152–3434.84)	3200.83 ± 492.27 (2888.05–3513.61)	0.56
Apgar score 5 min (mean, standard deviation, and 95%CI)	8.57 ± 0.69 (8.24–8.91)	8.75 ± 0.75 (8.27–9.22)	0.73
Gender (*n*/%)	Male = 11 (57.89%)	Male = 6 (50%)	0.47
	Female = 8 (42.11%)	Female = 6 (50%)	
Mode of delivery (*n*/%)	Cesarean = 6 (31.58%)	Cesarean = 4 (33.33%)	0.61
	Vaginal = 13 (68.42%)	Vaginal = 8 (66.67%)	
Premature rupture of membranes (*n*/%)	No = 18 (94.74%)	No = 11 (91.67%)	0.63
	Yes = 1 (5.26%)	Yes = 1 (8.33%)	
Preterm labour (*n*/%)	No = 17 (89.47%)	No = 11 (91.67%)	0.67
	Yes = 2 (10.53%)	Yes = 1 (8.33%)	

ICU—intensive care unit, CI—confidence interval.

## Data Availability

The data presented in this study are available on request from the corresponding author. The data are not publicly available due to local policies.
